# Knowledge and Awareness of Systemic Lupus Erythematosus Among the Population of Makkah, Saudi Arabia: A Cross-Sectional Study

**DOI:** 10.7759/cureus.41294

**Published:** 2023-07-03

**Authors:** Zuhair O Radhwi, Bassam M Bin Laswad, Abdullah A AlSulami, Faris Y Bahakeem, Mohammed A Abu zabiba, Siraj M Bawazer, Abdullah A Bajodah, Yusuf M Khairo, Waleed A Hafiz

**Affiliations:** 1 Department of Medicine and Surgery, College of Medicine, Umm Al-Qura University, Makkah, SAU; 2 Department of Medicine, College of Medicine, Umm Al-Qura University, Makkah, SAU; 3 Department of Medicine, Alnoor Specialist Hospital, Makkah, SAU

**Keywords:** health promotion, saudi arabia, general population, awareness, knowledge, rheumatology, autoimmune disease, systemic lupus erythematosus

## Abstract

Background

Systemic lupus erythematosus (SLE) is a multisystem autoimmune disease that affects mostly females of childbearing age. The exact cause is still unknown, but hormonal and immunological features and genetic predisposition are considered likely etiological factors. Disease presentation is variable but is usually characterized by phases of remission and relapse.

Objective

This study was conducted to assess the knowledge and awareness of SLE among the general population of Makkah, Saudi Arabia.

Methods

Data were collected using an online survey created with Google Forms that was distributed to the residents of Makkah aged 18 years and older. The survey was disseminated between November 2022 and January 2023.

Results

A total of 488 participants were included in the study, and the mean age of the participants was 29.1 years; the majority were female (54%). Only 18.6% of the participants knew someone with SLE, while 52.3% had heard about SLE. Additionally, 79.9% of participants had below-average awareness of SLE. Moreover, 72.1% believed that awareness of SLE should be promoted; 65.5% knew about SLE from the Internet or e-resources. Finally, only 5.5% of the participants had heard about SLE from physicians.

Conclusion

This study showed an insufficient level of knowledge and awareness of SLE in the general population of Makkah. We recommend conducting physical and virtual disease awareness campaigns and activities to enhance the knowledge and awareness of SLE among the general population of Makkah and other parts of Saudi Arabia.

## Introduction

The immune system is one of the most important systems in living organisms, generally discriminating between friend and foe [1]. Although the immune system is designed to protect an organism, such as a human, it can sometimes cause harm by attacking the organism’s own molecules, a phenomenon called autoimmunity [2]. The causes of autoimmunity remain largely unknown, but several studies have shown associations with environmental and genetic factors as well as certain types of infections [3-5]. Diseases that are caused by autoimmunity, known as autoimmune diseases, include rheumatoid arthritis, multiple sclerosis, type 1 diabetes, myasthenia gravis, and systemic lupus erythematosus (SLE), the latter of which is the focus of this study [2].

SLE is a chronic inflammatory autoimmune disease in which the immune system attacks the body’s tissues and organs [6]. It is distinguished by abnormal inflammatory responses as a result of complicated, aberrant humoral and cellular immune responses [3-5]. The combination of genetic predisposition with environmental, immunological, and hormonal variables leads to the clinical development of SLE, which has significant prominence among females of reproductive age [7-9]. Moreover, in all age and ethnic categories, SLE is more prevalent in females than in males [6]. Multiple autoantibodies that result in the development and deposition of immune complexes, as well as other immunological processes, have been linked to the symptoms of SLE [10]. Any organ can be impacted, including cardiovascular, respiratory, hematologic, renal, neurological, and skin organs, as well as having psychiatric effects [7]. Although SLE is a classic autoimmune condition, its frequency is estimated to be between 6.5 and 178.0 per 100,000 people, with an annual incidence between 0.3 and 23.7 per 100,000 people [11]. Management of SLE slows down the disease’s development and prevents new sequelae. However, at present, there is no single treatment option for SLE. When it is initially diagnosed, it exhibits high activity. Currently, the cornerstone of treatment is the use of glucocorticoids and disease-modifying anti-rheumatic drugs (DMARDs) to achieve and maintain remission [12,13].

In India, a survey conducted in 2017 to evaluate general community awareness and understanding of SLE revealed that most participants knew little about the condition as a rare disease affecting the populace [14]. Another study in Al-Hassa, Saudi Arabia, in 2018 showed that university students had low awareness and some misunderstandings regarding SLE [15].

To the best of our knowledge, no such study has been conducted in the city of Makkah. Thus, our study’s aim was to investigate the knowledge and awareness of SLE in the general population of Makkah, Saudi Arabia.

## Materials and methods

Study design and participants

A survey-based cross-sectional study was conducted among residents of Makkah, Saudi Arabia, to assess their knowledge and awareness of SLE. The study included males and females over the age of 18 living in Makkah who agreed to participate; participants with a diagnosis of SLE or having a first-degree relative diagnosed with SLE were excluded. Additionally, healthcare practitioners were excluded from the study.

Ethical considerations and sample size

A self-administrated electronic questionnaire created through Google Forms was distributed through social media platforms from November 2022 to January 2023. The required sample size was calculated through OpenEpi, version 3.01, and the minimum required sample was found to be 385 participants, keeping the confidence interval at 95% [16]. A convenience sampling technique was used to recruit participants. The study was approved by the Umm Al-Qura University Biomedical Ethics Committee (approval number: HAPO-02-K-012-2022-11-1298).

Study tool

We adopted a validated assessment tool from a previously published study [17]. The questionnaire was translated into Arabic, and then a pilot study was conducted to assess the validity of the Arabic version of the questionnaire. The data from the pilot study were excluded from the final dataset used in the study.

The questionnaire sections included sociodemographic characteristics and questions regarding knowledge and awareness of SLE. Data were collected after obtaining participants’ consent. Contact information was attached to the questionnaire to answer any inquiries.

Statistical analysis

Data were collected, reviewed, and then fed into IBM Statistical Package for Social Sciences (SPSS) version 26.0 (Released 2019; IBM Corp., Armonk, New York, United States). All statistical methods used were two-tailed with an alpha level of 0.05; significance was considered if a P-value was less than or equal to 0.05. For participants’ awareness, each correct answer was given a score of one point. An overall awareness score of SLE was assessed by summing up discrete scores for different correct knowledge items. If the total score was above the mean score value, the level of awareness was considered to be good (aware), and a score less than the mean value was considered poor (unaware).

Descriptive analysis was done by prescribing frequency distribution and percentage for each study variable, including participants’ personal data, knowing others with SLE, and source of information. Furthermore, participants’ awareness of SLE was tabulated, while their overall awareness levels and the sources of their information were graphed. Cross-tabulation was done to show the distribution of participants’ overall awareness levels according to their personal data and other factors using Pearson’s chi-square test for significance and an exact probability test if there were small frequency distributions.

## Results

A total of 488 participants fulfilling the inclusion criteria were included in the study and completed the questionnaire. Participants’ ages ranged from 18 to 68 years, with a mean age of 29.7 ± 11.8 years. A total of 266 (54.5%) participants were female, 238 (48.8%) were students, and 129 (26.5%) were employed. In terms of education, 364 (74.6%) were university graduates, 33 (6.8%) had post-graduate degrees, and 11 (2.3%) had an educational level below the secondary level. A total of 188 (38.5%) were married, while 284 (58.2%) were single. A monthly income of less than 5,000 Saudi Riyal (SR) was reported among 116 (23.8%) participants, 155 (31.8%) had a monthly income of 5,000-9,000 SR, and 42 (8.6%) had a monthly income exceeding 10,000 SR (Table 1).

**Table 1:** The demographic data of study participants (n=488) SR: Saudi Riyal

Variable	No	%
Age, in years		
18-20	112	23.0%
21-25	157	32.2%
26-30	47	9.6%
31-39	68	13.9%
40+	104	21.3%
Gender		
Male	222	45.5%
Female	266	54.5%
Employment status		
Unemployed / retired	121	24.8%
Student	238	48.8%
Governmental employee	96	19.7%
Non-governmental employee	33	6.8%
Education level		
Below secondary	11	2.3%
Secondary	80	16.4%
University	364	74.6%
Post-graduate	33	6.8%
Marital status		
Single	284	58.2%
Married	188	38.5%
Divorced / widow	16	3.3%
Monthly income		
< 5000 SR	116	23.8%
5000-9000 SR	155	31.8%
10000-20000 SR	175	35.9%
> 20000 SR	42	8.6%

Only 18.6% of participants knew someone who had been diagnosed with SLE; 52.3% had heard about the disease; and a solitary 6.8% knew that SLE is contagious. Regarding disease severity, 17.6% reported that it is of moderate risk (not fatal), while 11.7% believed that SLE is risky (may be fatal), and 8% believed it is fatal. A total of 35.2% knew that SLE is an autoimmune disease with no known cause, and 16.8% indicated that SLE mostly affects females. Meanwhile, 26.6% reported that SLE affects any organ or part of the body, with the most reported being the skin (27.5%), joints (16.8%), kidneys (14.1%), heart (11.3%), and blood (11.3%). The most well-known symptoms of SLE among study respondents were rashes (35.2%), joint pain (21.9%), alopecia (14.1%), and hematuria (8%). Of the participants, 16.2% knew that SLE cannot be diagnosed with a single blood test, 22.1% believed that SLE can be prevented, 6.6% reported that SLE is an illness with few complications, and 31.1% agreed that SLE is a treatable disease. Regarding treatment methods, 8.2% indicated steroids, 3.1% chose chemotherapy, 1.2% selected malaria medications, and 12.1% chose a combination of these medications. Additionally, 8.8% believed that SLE may affect the fertility of men and women, and 17.8% agreed that SLE causes fetal abnormalities or recurrent abortions in the affected mother. A total of 72.1% believed that awareness of SLE should be promoted (Table 2).

**Table 2:** Participants’ awareness of SLE, frequencies, and percentages (n=488) SLE: systemic lupus erythematosus

Questions		No	%
Do you know someone who has been diagnosed with SLE?	None	397	81.4%
	Friends	44	9.0%
	Relative	38	7.8%
	Others	9	1.8%
Have you heard about SLE?	Yes	255	52.3%
	No	233	47.7%
Is SLE contagious?	Yes	33	6.8%
	No	154	31.6%
	Don't know	301	61.7%
What do you think is the severity of SLE?	Not dangerous	11	2.3%
	Simple risk	7	1.4%
	Moderate risk (not fatal)	86	17.6%
	Risky (may be fatal)	57	11.7%
	High-risk (fatal)	39	8.0%
	Don't know	288	59.0%
What is the nature of SLE?	There is no cause (the body fights itself)	172	35.2%
	It's hereditary	103	21.1%
	There are tumors	29	5.9%
	Don't know	290	59.4%
Does SLE mostly affect females?	Yes	82	16.8%
	No	27	5.5%
	Don't know	379	77.7%
Does SLE affect any organ/part in the body?	Yes	130	26.6%
	No	45	9.2%
	Don't know	313	64.1%
Which organs can be affected by SLE?	Skin	134	27.5%
	Joints	82	16.8%
	Kidney	69	14.1%
	Heart	55	11.3%
	Blood	55	11.3%
	Liver	51	10.5%
	Lungs	40	8.2%
	Eyes	35	7.2%
	Don't know	289	59.2%
Which of the following are symptoms of SLE?	Rash	172	35.2%
	Joint pain	107	21.9%
	Alopecia	69	14.1%
	Hematuria	39	8.0%
	Photosensitivity	39	8.0%
	Don’t know	280	57.4%
Can SLE be diagnosed with a single blood test?	Yes	40	8.2%
	No	79	16.2%
	Don't know	369	75.6%
Can SLE be prevented?	Yes	108	22.1%
	No	40	8.2%
	Don't know	340	69.7%
Is SLE a treatable disease?	Yes	152	31.1%
	No	55	11.3%
	Don't know	281	57.6%
Is SLE an illness with few complications?	Yes	32	6.6%
	No	106	21.7%
	Don't know	350	71.7%
Which of the following is a treatment for SLE?	Chemotherapy	15	3.1%
	Steroids	40	8.2%
	Malaria medications	6	1.2%
	Combination of the above medications	59	12.1%
	Others	10	2.0%
	Don’t know	358	73.4%
Can SLE affect the fertility of men and women?	Yes	43	8.8%
	No	34	7.0%
	Don't know	411	84.2%
Can SLE cause fetal abnormalities or recurrent abortions in the affected mother?	Yes	87	17.8%
	No	20	4.1%
	Don't know	381	78.1%
Should awareness of SLE be promoted?	Yes	352	72.1%
	No	7	1.4%
	Don't know	129	26.4%

**Figure 1:**
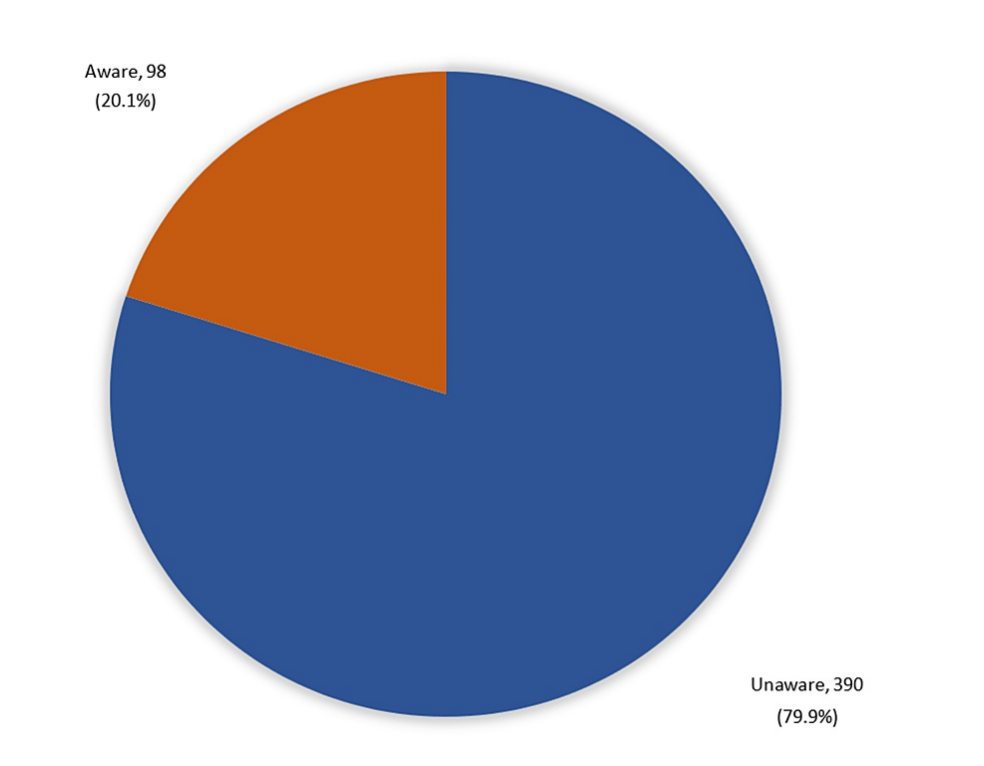
Participants’ overall awareness regarding SLE (n=488) SLE: systemic lupus erythematosus

A total of 98 (20.1%) participants were aware of SLE, while 390 (79.9%) had a below-average awareness level (Figure 1).

**Figure 2:**
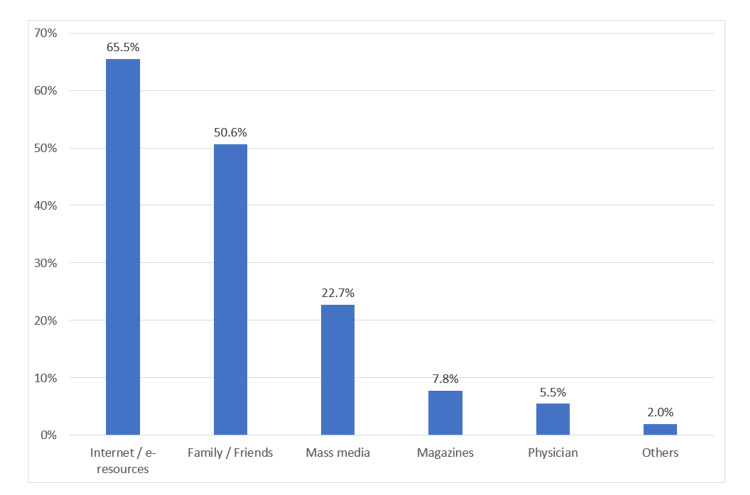
Sources of information regarding SLE among study participants (n=488) SLE: systemic lupus erythematosus

The most reported source of knowledge and awareness of SLE reported by the respondents was the Internet/e-resources (65.5%), followed by family/friends (50.6%), mass media (22.7%), magazines (7.8%), and physicians (5.5%) (Figure 2).

Of the participants, 26.3% of the females had good awareness levels versus 12.6% of the males, with a recorded statistical significance (P=.001). Also, 26.1% of the students had good awareness levels, compared to 12.1% of those working in the non-governmental sector. Good awareness was detected among 34.1% of participants with friends diagnosed with SLE compared to 17.9% of others without (P=.049). All other factors showed an insignificant relationship with participants’ awareness levels (Table 3).

**Table 3:** The relationship between the awareness level of SLE and participants' demographic characters (n=488) SLE: systemic lupus erythematosus; SR: Saudi Riyal * P<0.05 (significant); $: exact probability test

Factors	Overall awareness level				P-value
	Unaware		Aware		
	No	%	No	%	
Age, in years					
18-20	80	71.4%	32	28.6%	0.092
21-25	127	80.9%	30	19.1%	
26-30	40	85.1%	7	14.9%	
31-39	54	79.4%	14	20.6%	
40+	89	85.6%	15	14.4%	
Gender					
Male	194	87.4%	28	12.6%	0.001*
Female	196	73.7%	70	26.3%	
Employment status					
Unemployed/retired	103	85.1%	18	14.9%	0.015*
Student	176	73.9%	62	26.1%	
Governmental employee	82	85.4%	14	14.6%	
Non-governmental employee	29	87.9%	4	12.1%	
Education level					
Below secondary	8	72.7%	3	27.3%	0.915
Secondary	63	78.8%	17	21.3%	
University	292	80.2%	72	19.8%	
Post-graduate	27	81.8%	6	18.2%	
Marital status					
Single	220	77.5%	64	22.5%	0.273
Married	157	83.5%	31	16.5%	
Divorced / widow	13	81.3%	3	18.8%	
Monthly income					
< 5000 SR	91	78.4%	25	21.6%	0.945
5000-9000 SR	123	79.4%	32	20.6%	
10000-20000 SR	142	81.1%	33	18.9%	
> 20000 SR	34	81.0%	8	19.0%	
Do you know someone who has been diagnosed with SLE?					
None	326	82.1%	71	17.9%	0.049*
Friends	29	65.9%	15	34.1%	
Relative	29	76.3%	9	23.7%	
Others	6	66.7%	3	33.3%	
Source of information regarding SLE					
Internet/e-resources	105	62.9%	62	37.1%	0.386^$^
Physician	8	57.1%	6	42.9%	
Family/Friends	92	71.3%	37	28.7%	
Mass media	37	63.8%	21	36.2%	
Magazines	13	65.0%	7	35.0%	
Others	3	60.0%	2	40.0%	

## Discussion

SLE is an autoimmune condition in which the body produces antibodies directed against nuclear and cytoplasmic antigens, affecting several organs such as the skin, kidneys, joints, and the central nervous system. SLE usually affects females between puberty and menopause, but it can also affect children in 15% to 20% of cases; the latter has a more aggressive disease course than adult-onset SLE. Signs and symptoms of SLE can include fever, fatigue, dry eyes, and a butterfly-shaped rash on the face that covers the cheeks and bridge of the nose; the severity of the disease can vary from mild to life-threatening [18-20].

Since SLE patients have a three-times higher mortality risk than the general population, awareness of the disease is important [21]. Thus, our study aimed to assess the awareness and knowledge of SLE and its pathophysiology, epidemiology, complications, severity, and treatments among the general population of Makkah.

Our study results demonstrated that most of the studied participants (79.9%) had a poor level of knowledge and awareness regarding SLE, and this finding concurred with different studies conducted in different regions in Saudi Arabia [17,22,23].

In the present study, 488 participants were selected after meeting the inclusion criteria, with 45.5% male and 54.5% female participants, a near-equal gender distribution. On the other hand, a similar study conducted in the city of Abha included 33.3% male and 66.7% female participants, and another study in Riyadh included 41.3% male and 58.7% female participants [17, 22].

We asked participants about the nature of the disease and found that 35.2% believed that it is an autoimmune disease, 21.1% believed that it’s a hereditary disease, 5.9% believed that it involves tumors ("there are tumors"), and 59.9% said they did not know. In comparison, in the study conducted in Riyadh, 25.8% believed that it is an autoimmune disease, 29.8% believed that it’s a hereditary disease, 16% believed that "there are tumors," and 27.4% said they "don’t know" [17]. The variation in the results between the two samples may be attributed to the fact that in Makkah, there are yearly campaigns directed at the public about rheumatological diseases. But surprisingly, the majority of our study population (77.7%) couldn’t recognize that SLE affects females predominantly. Furthermore, only 8.8% of the studied participants clarified that SLE can affect fertility in both males and females.

Upon exploring the influence of participants’ sociodemographic characteristics on awareness levels, we found that females had a higher awareness level in comparison to male participants, with recorded statistical significance (P=0.001). This was expected since SLE is more prevalent among females. In comparison, the study conducted in Riyadh discussed above showed that the difference in awareness between males and females was insignificant (P=0.304) [17]. Meanwhile, in the study conducted in Abha [22], they obtained unexpected results, finding that awareness levels among males were higher than among females in their sample (P=0.018).

Furthermore, our study revealed that students had a significantly higher awareness level in comparison to those who were unemployed or retired, governmental employees, and non-governmental employees, with recorded statistical significance (P=0.015). This placed students in this study as the most aware compared to the other groups in the study. In Asiri et al.'s study, they found that medical professionals were the group with the highest awareness levels when compared to the other groups in their study [22]. The reason that may have led to the students being the highest group in awareness level is their exposure to colleagues from medical colleges in the university, unlike the unemployed or retired participants who may not have been able to complete their Bachelor’s degree. As for the employed participants, it may be attributed to the fact that they do not have enough free time; the solution to that may be through targeted campaigns in the workplace.

Finally, we found that participants who knew someone diagnosed with SLE had a higher level of awareness (P=0.049). This was expected since levels of awareness should be higher among those who know someone with SLE. In the study conducted in Riyadh [17], they found that participants knowing someone who has been diagnosed with SLE was not a key factor in the general awareness levels regarding SLE, since the group with the highest awareness levels were the people with no association with anyone who has been diagnosed with SLE.

Limitations

Since this study is limited to the population of Makkah, it may be difficult to generalize the results. Also, since the data were obtained via an online questionnaire, the study was limited to a stratum of the population with access to the Internet. Recall bias could also be possible.

## Conclusions

This study shows that the knowledge and awareness of SLE among the general population of Makkah are poor. Thus, education about the disease, its clinical signs and symptoms, complications, severity, and treatment through public awareness campaigns, social media, social media influencers, and e-brochures is necessary. Furthermore, we recommend conducting more studies in other regions of Saudi Arabia to assess the levels of knowledge and awareness of SLE throughout the kingdom.
